# Idea Generation and New Direction for Exploitation Technologies of Coal-Seam Gas through Recombinative Innovation and Patent Analysis

**DOI:** 10.3390/ijerph17082928

**Published:** 2020-04-23

**Authors:** Lijie Feng, Yilang Li, Zhenfeng Liu, Jinfeng Wang

**Affiliations:** 1 School of Management Engineering, Zhengzhou University, Zhengzhou 450001, China; liyilang152983@gs.zzu.edu.cn (Y.L.); wangjinfeng@shmtu.edu.cn (J.W.); 2 School of Economic & Management, Shanghai Maritime University, Shanghai 201306, China; zfliu@shmtu.edu.cn

**Keywords:** coal-seam gas, idea generation, recombinative innovation, patent analysis

## Abstract

Coal-seam gas (CSG), as an alternative energy, has the characteristics of resource scarcity and technological exploitation complexity. The generation of ideas is vital to develop more efficient exploitation technologies for CSG. Innovative ideas originate from the recombination of existing knowledge elements according to recombinative innovation. The previous literature has focused on exploring an abundance of combinations, which leads to blindness towards idea generation. For this reason, it is critical to search for more valuable matching patterns among the redundant combinations of elements. In line with this concept, this paper proposes a method that consists of three phases: the collection of knowledge elements, the analysis of knowledge element depth and diversity, and the analysis of knowledge element relationships. In this process, we take the patent document as the carrier of knowledge recombination and identify the optimization method in the reorganization process by means of latent Dirichlet allocation (LDA) and association rules. This method is expected to assist in sparking better ideas for CSG exploitation technologies.

## 1. Introduction

Coal-seam gas (CSG) is a naturally occurring methane gas found in most coal beds. The demand for CSG has been driven by a combination of factors, including the impetus to develop alternatives to fossil fuels (e.g., coal and oil), the heavy smog in China and India, and increasing concerns about global climate change [1]. Exploitation technologies for CSG are critical to the increased demand for gas and even a lower carbon future [2]. Instead, the inefficient exploitation technologies for CSG have resulted in China’s coal mines accounting for more than half of global emissions of CSG [3,4]. It is worth studying the problem of how to develop more exploitation technologies suitable for different coal seams. A solution to overcome this problem is generating ideas for recombinative innovation. The reasons to solve this problem involve three aspects. First, CSG exploitation is a complex technology involving multiple disciplines, such as geology, mechanics, materials, and machinery. Currently, increasing technological complexity has been accompanied by recombinative innovation, which has been studied by many scholars [5,6,7,8,9,10,11]. Second, from the perspective of energy scarcity and rapid innovation, it is believed that the technologies needed for CSG can be discovered and the existing knowledge elements can be recombined by use of recombinative innovation. Third, to innovators, recombinative innovation not only plays a critical role in the maintenance of sustained technological advantages, but also saves the time and cost of developing a technology [6]. According to Schumpeter’s recombinative innovation, it is generally accepted that most innovative ideas can be obtained by recombining existing knowledge elements [12,13,14]. By following his theory, a plethora of studies in the technological innovation literature have come to a convergence indicating that an idea can be regarded as a set of technical knowledge elements and that the generation of creative ideas can be achieved by reorganizing these knowledge elements [9,15,16,17]. In the process of recombinative innovation, depth and diversity, as the basic characteristics of knowledge elements, are particularly important [18]. However, the previous literature has regarded the knowledge base involved in a technical system as a simple superposition of knowledge elements and thus has focused on the relationship between the quantitative characteristics of knowledge elements and the innovation results [14,19]. Furthermore, it is rarely discussed how the different knowledge elements are linked together in a coupling way. These relations can explain the fashion in which those knowledge elements are combined, and hence also provide a channel for exploring the heterogeneous reorganization of knowledge elements to create new combinations of knowledge elements [20,21]. The coupling relationship between knowledge elements can be explored by means of data mining technology. One of the most important data mining technologies is association rule mining. Its most important purpose is to discover all the important associations between different items in the data store [22].

Another solution to overcome the research problem is an analysis of CSG patents. Since patent documents often contain important technical elements concerning a technological field [23,24], they can be used as a source of technical knowledge for generating new combinations to obtain new ideas [17,25]. To date, previous studies have identified technology clusters based on patents or term association. However, these studies have limitedly assumed a patent belongs to only one technological subject, while in fact, it often belongs to multiple subjects. Consequently, existing methods have failed to analyse potential technical topics contained in the text of patents. In addition, most studies have analysed the clusters of primary or emerging technologies without considering their opportunity potential. Hence, examining the potential of technical topics based on their importance and satisfaction can provide insights into research and development [26]. Therefore, clarifying the innovation topics and their characteristics can help technical experts deeply understand the knowledge elements contained in a technology [27]. In terms of identifying the diversity of knowledge elements, since the characteristics of complex problems are often multidimensional and uncertain [28], it is necessary to introduce a technical classification system to conduct the multidimensional analysis of knowledge elements with different attributes. Based on this concept, this paper uses the latent Dirichlet allocation (LDA) algorithm to extract the technical topics from patent text and related technical keywords and combines the specific technical classification system to realize the deconstruction of knowledge elements. Specifically, this paper uses the LDA algorithm to identify and analyse the subjects in the target technology field and mine the technical keywords corresponding to these subjects, which are different from high-frequency keywords that can reflect the technical characteristics of the target field to a certain depth. At the same time, by combining with the existing technology classification system TEMPEST (thing, energy, material, personality, space, and time), the multidimensional analysis of knowledge elements is carried out. TEMPEST classifies the diversity characteristics of these knowledge elements according to technical attributes, so it is helpful to create new ideas [29]. The second step is to explore the relationship between the knowledge elements. In this step, association rules first analyse the relationship between knowledge elements of different dimensions, to grasp the association strength of knowledge elements in different coupling ways and screen out more potential element combinations from the many combination ways. Then, domain experts judge whether the different improvement directions in SCAMPER (substitute, combine, adapt, modify, put to other use, eliminate, and rearrange) can be applied to these combinations and further use the association rules to identify the promotion degree of different improvement ways under specific coupling paths. Finally, the promotion degree is used as a judgment standard to trigger the priority of creative generation, helping researchers to provide the direction of idea generation.

In summary, for the recombinative innovation and patent analysis of CSG, this paper designs a set of system processes that can realize the combination of qualitative modelling and quantitative analysis, which has improved the degree of automation in the process of traditional idea generation. Because of the priority identification in the analysis of the coupling mode of knowledge elements, R & D personnel can trigger new ideas in a directional way during the process of generating ideas for CSG to overcome the blindness of creative generation to a certain extent. The overall structure of this paper is as follows. Section 2 reviews related studies on the generation of ideas and recombinative innovation, and relevant algorithms for patent text mining are also introduced, including the LDA topic extraction algorithm and association rule algorithm. Section 3 explains our methodology in detail, and a case study is presented in Section 4. Finally, in Section 5, we conclude this paper and identify the contributions and limitations.

## 2. Background

### 2.1. Idea Generation Methods

In many cases, idea generation is conducted by using the experts’ knowledge. However, the use of idea generation methods seems to have increased and fostered the creative ideation process. With systematic methods, one can consider more thoroughly the causes of problems and find new solutions in a systematic manner [30]. As a result, there is considerable literature devoted to new ways of generating ideas [31].

Among the many methods for fostering creativity, morphology analysis is considered a powerful systematic methodology [31,32,33]. The strength of morphology analysis lies in its ability to model complex problems in a non-quantitative manner. A system typically consists of a synthesis of a number of separate subsystems, each of which may be realized in a number of different ways [34]. In this sense, morphology analysis provides a strong advantage to structuring and analysing technological, organizational and social problems by breaking the subject down into a number of fundamental dimensions [34,35]. TRIZ (the theory of inventive problem solving) claims that difficult problems can be codified, classified, and solved methodically, just like other engineering problems. The check list method is a kind of creative technique with an ideal effect, which is mainly used in the research and development of new products and focuses on guiding people to solve problems according to various ideas of check projects. The SCAMPER model is a method developed by Alex Osborn and Bob Eberle to stimulate the generation of new ideas [25]. SCAMPER is an abbreviation of seven different words, namely S (Substitute), C (Combine), A (Adapt), M (Modify, Minify, Magnify), P (Put to other use), E (Eliminate), R (Rearrange, Reverse). SCAMPER represents seven directions that can improve or change the technical system during technological innovation.

In Table 1, we make a comparison and summary of idea generation methods. Starting from the nature of recombinative innovation of knowledge elements, we can have a further understanding of traditional innovation methods. For example, after constructing the morphological matrix, the elements are recombined to stimulate new ideas or technical solutions. In the process of idea generation or technological innovation, first of all, different innovative methods focus on the characteristics of the technical system itself, such as the form, rudiments, and functions of knowledge elements, which can be understood as a dimensional analysis of the technical system itself. On this basis, traditional innovation methods generate new technological forms or ideas by redistributing the relationship between knowledge elements. 

It is worth noting that the purpose of this paper is not to replace traditional idea generation methods. However, traditional creative generation methods usually ignore the search for higher priority ideas in the process of generating ideas. Since the various combinations are not prioritized, this will lead to the accumulation of numerous combinations, so there will be confusion when stimulating researchers to generate new ideas. In response, the focus of this paper is to embed the idea of recombinative innovation into the process of idea generation and to explore the combination of factors with higher priorities starting from the diversity of knowledge elements and the type of coupling between elements. At the same time, with the help of scientific data analysis tools, the approach helps trigger R & D personnel to form better ideas based on these combinations from a large number of choices, thereby improving decision-making efficiency.

### 2.2. Recombinative Innovation

First, recombination needs to find unexplored interdependencies of diverse knowledge elements within the actors’ purview [6,36]. Fleming [37] proposed that technology classification can be used to illustrate the process of technology recombination. Technology classification is effective and widely used to express patented technology components [6,38]. The patent classification method was first introduced in the previous study and involves three methods. One method is the international patent classification (IPC). Specifically, in these attempts, the IPC system or a set of keywords, which are identified using a text mining application with patent documents, are usually used for opportunity analysis [29]. These studies have their merits, while they also have some disadvantages. First, technical opportunity analysis using IPC may lack specificity because it seems too simple to classify technologies through a single IPC view to represent all aspects of a technology. The other method is F-term, which is a patent classification system that classifies patent documents according to the technical attributes of the invention. Because technical attributes are analysed from different perspectives, it is possible to investigate a wider range of technologies than other classification systems [39]. By using F-term, technical attributes are analysed from different perspectives, and the technical information provided by F-term is more detailed than that provided by the IPC system and more systematic than that provided by keywords [29]. Another method used for patent classification is TEMPEST, which first introduced the patent classification method in previous research and is defined as one of the methods to classify patents from different perspectives and for the qualitative analysis of technical information [25]. Each letter of TEMPEST (E, M, P, S, and T) stands for a personal opinion that analyses patents. Based on the view of E (energy), the corresponding patent includes technical information, mainly relating to the drive device or the way the drive device works. From the point of view of materials, the characteristics of a patent are materials composed of products or technologies, i.e., raw materials and substances. A developed technology/product naturally has its unique role, which may be called function, and then attempts to generate the attributes of the technology in P (personality) [25]. Each letter, E, M, P, S, and T, in the TEMPEST model represents the analytical perspectives of different dimensions in the analysis of patents. It is worth noting that P (personality) involves two important features of the function and mechanism of the technical system, so P is further decomposed into two analytical perspectives of F (function) and M (mechanism). The specific contents are shown in Table 2.

To solve these problems, this study adopts the TEMPEST model, which classifies patents according to the detailed technical attributes of functions, structures, materials, operating procedures and so on [29]. By using technical attributes rather than technical content, technologies with similar invention principles can be identified, enabling combinations of heterogeneous elements, which will facilitate the ideation process for idea generation in specific technical areas.

### 2.3. Patent Analysis

In recent years, text mining, as a method to extract valuable information from an abundance of unstructured text, has been widely used to explore the complex relationship between patent documents [40]. Most text mining tools assume that keywords can be used to label the important content of documents [41], and thus the operation for knowledge discovery can be executed with the labels of documents. The true value of patent analysis stems from its capability of describing the content of technology based on the relationships between keywords [42]. Some words that can reflect the technical characteristics of sensitive information appear in the text at a low frequency, which will cause most words that can represent the text to be drowned by some high-frequency words [43]. Many patent analyses employ k-means clustering [44,45,46]. The efficiency of the k-means clustering algorithm increases with the clustering distance. However, marking a document with a single word does not guarantee the complete separation of set documents under certain conditions due to noise, and the problem of data separation still exists. K-means clustering is not an appropriate analytical tool [47]. Despite the contributions of these studies, more attention has been paid to the study of technical keywords, which shows limitations in the analysis of technological innovation themes.

Previous studies have used clustering technology based on patents or term association to identify technology clusters but have failed to identify and analyse the potential technical topics with multiple patents or terms. Examining the potential of technical topics based on their importance and satisfaction can provide insights for research and development [26]. The purpose of topic modelling is to discover topics that span document collections. A convincing topic model can help extract an accurate understanding of key information from a large corpus [48]. With the rising amount of text data and the in-depth study of machine learning, the method of automatic discovery of potential topics in documents based on a topic model is becoming increasingly popular. Specific algorithms differ from one another, but topic models typically assume that a topic contains a set of words that often appear together. In other words, texts on different topics may use different vocabularies [27]. LDA is widely used in many topic modelling methods and is the most typical topic model [43]. LDA is essentially a Bayesian thematic model, which has been widely used in recent years. Basically, LDA follows a probability distribution model, which assumes that writing is the process of selecting subject words [49]. Based on this assumption, the LDA represents the document as a hybrid model containing K potential topics, with each topic as a multinomial distribution of words in the document [49,50]. The problem with determining a document topic is that you can only observe the document, but the topic structure is hidden (such as the topic itself, the topic distribution within the document, the word distribution per topic, and the topic assignment per word within each document). LDA solves this problem by tracing back the observed word distribution in a given document to infer hidden topic structures that might generate a collection of documents. From the computational point of view, inference means to calculate the conditional distribution of implicit variables of a given document, which is called a posterior distribution in Bayesian language. LDA uses a word package approach (the word bag model) that treats each document as a word frequency vector, transforming textual information into easily modelled digital information. Each document represents the probability distribution of the topic, and each topic represents the probability distribution of multiple words.

In these data mining techniques, association rule mining is an effective analysis technique because the data mining method does not rely on any assumptions, and can find meaningful connections hidden in large data sets [51]. Association rules are based on a statistical analysis and artificial intelligence [52]. By considering the conditional interaction between input data sets, this technique extracts frequent item sets, satisfies the parameters such as minimum support degree and minimum confidence degree, and generates association rules, which is particularly suitable for the study of the relationship between different dimensions. Association rules are designed to provide insight and sufficient knowledge for decision makers to make correct optimization decisions based on existing data sets. This approach consists of two main steps: The classic Apriori algorithm is used to extract association rules, and then, through a multicriteria decision analysis, the interested rules are evaluated and selected from the extracted strong association rules. In this study, we use the Apriori algorithm to discover important rules between knowledge elements, explore the best matching patterns of factor reorganization, and provide guidance for the generation of ideas.

## 3. Methodology

### 3.1. Overall Research Framework

**Figure 1:**
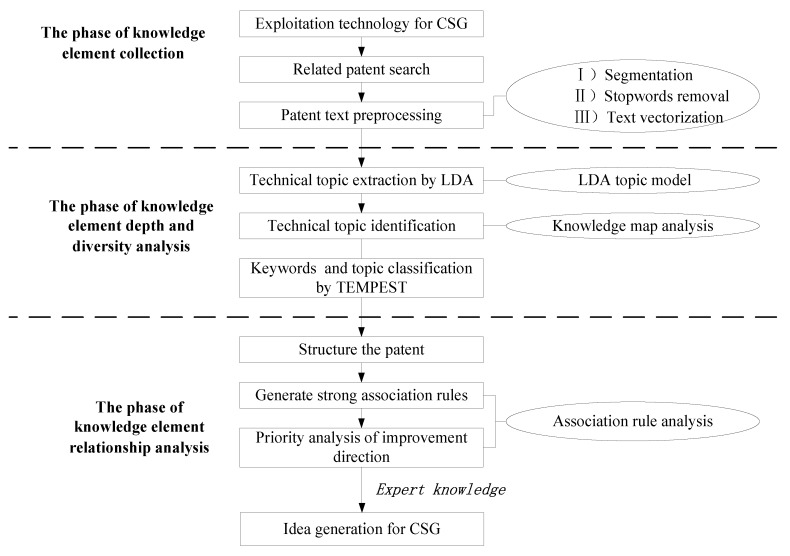
Research framework.

Through the analysis of recombinative innovation, new technology opportunities or the emergence of new ideas often lies in the restructure of existing knowledge elements, and this study will reorganize the process of further analysis on the characteristics of the knowledge factors. The first characteristic is the depth of the knowledge itself and the diversity of property. Another characteristic is the method of coupling between elements. These two important characteristics strongly affect the quality of the generated new ideas. Based on this notion, embedding the idea of reorganization innovation into the process of creative generation can provide a guiding path for the finding more potential new ideas. As shown in Figure 1, our proposed method consists of three phases: the knowledge element collection, the knowledge element depth and diversity analysis, and the knowledge element relationship analysis. The first phase is mainly designed to collect and preprocess patent data. Since patent text contains rich knowledge elements, several knowledge elements can be obtained through an extensive patent search. In the second phase, the first feature of knowledge elements will be analysed. In this paper, the LDA model, a text analysis tool, will be used to identify the technical topics reflected in patent texts and the technical keywords related to these topics. These technical keywords are the specific embodiment of the knowledge elements contained in the technical system, which can describe the technical features at a certain depth. At the same time, the LDA topic is identified by constructing a knowledge network map. Then, through the TEMPEST model, the technology topics and keywords are classified so that the knowledge elements of the technology system can be identified in diversity. The third phase will focus on exploring the relationship between the knowledge elements. In this process, the analysis of association rules is first used to screen out potential element combinations and then use the SCAMPER model to identify the improvement direction of each group combination. Further, through the support and lift to generate association rules and to test them, finally, we obtain the optimal improvement direction for a particular combination and can generate better ideas combined with expert experience.

### 3.2. Detailed Procedures

#### 3.2.1. The Phase of Knowledge Element Collection 

1. Select target technology and patent data collection

The first step is the collection of related patents. In this part, R & D personnel or companies collect relevant patents on CSG. The samples used for idea generation come from patents filed by the Chinese intellectual property office and the Derwent database between 2000 and 2018. The data source is appropriate for exploring recombinative innovation because it is a representative patent database containing an enormous number of patents from all over the world and covers the most advanced technologies [42].

In this study, the purpose of patent text collection is to further analyse the subject, function or technology reflected by selectively extracting patent parts that reflect technical characteristics. The beginning of collecting patents requires one to determine the scope, concept and purpose of the analytical task. In this process, the scope and type of the target technology system should be set, and the patent search query should be constructed by the keywords corresponding to these requirements. The process of querying and screening out patents related to tasks also requires the participation of domain experts to assist the determination of patent searching strategies. 

2. Patent text preprocessing

Since patents are usually semi-structured text formats, it is necessary to distinguish the structured and unstructured parts of them and save the structured parts for the subsequent analysis. In general, a complete patent text consists of different items, including headings, abstracts, the classification code, and so on. Among those items, the abstract of the patent documents describes the principle and the purpose of invention clearly and concisely, and the existing studies on patent analysis have made use of the abstracts of patents. In this paper, the abstract is used as the input to LDA to identify topics because it typically includes the main problem that the patented technology addresses and the core of the technological solution [27]. Therefore, in these unstructured abstracts, natural language processing is required to perform more preprocessing tasks, including tokenization, stop-word removal, and vector-space representation.

A word is the smallest meaningful language component that can act independently. Tokenization is the task of breaking a character sequence up into pieces (words/phrases) called tokens, and perhaps, at the same time, throw away certain characters such as punctuation marks. General tokenization is the first core technology of natural language processing. In this paper, every sentence of the abstract is broken into words. By doing this, a set of inflected forms of a word can be considered as a single item in the further analysis. The list of tokens then is used for further processing. Then, since function words such as prepositions, articles, and conjunctions are generally considered to provide no more useful value to the document, they are often added to a stop-word list and removed in the processing document. This step is called stop-word removal. Finally, the patent abstract is constructed as a keyword co-occurrence matrix, in which the determination of keywords in each abstract is jointly realized by TF-IDF and experts to prevent the omission of some specific technical keywords. The final result of text preprocessing is a vectorization matrix processed by natural language processing and used as the next input.

#### 3.2.2. The Phase of Knowledge Element Depth and Diversity Analysis

1. Technical topic extraction by LDA 

In this step, LDA-based topic modelling is adopted to identify the innovation topics portrayed in patents. Our topic modelling application requires two inputs: the patent keyword matrix and number of topics. The patent-keyword matrix obtained from the previous step can be used as the input matrix, while an appropriate number of topics should be determined for topic modelling.

**Figure 2:**
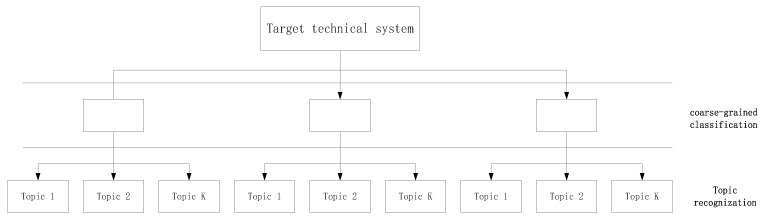
Sensitive information topic recognition framework.

The results of the subject distribution of LDA are characterized by keywords with different probabilities and the topics that reflect the distribution of patent texts without technical features in the relevant technical system field. The general framework for the LDA topic model is shown in Figure 2.

As shown in Figure 2, the topic recognition of sensitive information in patent data is divided into two steps. First, coarse-grained classification is carried out according to the distribution of keywords in the network knowledge map, and this step mainly depends on the analysis of the betweenness of keywords. Then, the LDA algorithm is used to identify and extract more detailed and sensitive information topics for the texts of each category.

2. Technical topics and keyword classification by TEMPEST

In this step, the identified topic is further explored. On the one hand, since these topics are supported by a series of specific knowledge elements, in this study, considering an important characteristic of recombinative innovation, the specific attributes of these knowledge elements need to be paid attention to. Therefore, a technology classification system is needed to classify the corresponding technical keywords on different dimensions. Specifically, this paper will conduct the dimensional analysis of the LDA clustering results through the TEMPEST model.

After the diversity and in-depth analysis of knowledge elements, we can obtain the knowledge elements of different dimensions, to further understand that a technical theme is formed by the collaborative services of knowledge elements of different dimensions. These knowledge elements are different from those reflected by high-frequency keywords and can deeply reflect the mechanism of the operation of the technical system, which is more conducive for R & D personnel.

#### 3.2.3. The Phase of Knowledge Element Relationship Analysis

1. Structure the patent and generate strong association rules

In the analysis phase of knowledge element relations, this paper explores the coupling relationship between knowledge elements through the analysis of association rules. Firstly, knowledge elements of different dimensions in a patent should be identified according to the analysis results of the knowledge elements. Specifically, the technical characteristics reflected by each patent are characterized by the corresponding technical keywords. In this paper, the results obtained by the LDA and TEMPEST models for each patent are compared to obtain the text format similar to the item set formed by the combination of different technical keywords. On this basis, this paper will filter out frequent item sets with an indicator of the support degree via association rules and explore the potential combination of closely related knowledge elements in the analysis of numerous projects as a guide for restructuring innovation.

Next, an important question to be answered is how to reconstruct these potential combination methods and how to judge the higher priority reconstruction methods from different reconstruction methods for idea generation. To this end, this paper will first improve the combination of knowledge elements through the idea generation method the scar model. Since the seven improvement directions related to innovative design in the SCAMPER model can provide guidance for R & D personnel, we can combine scar and expert experience to identify the possible improvements to the combination way, and the combination way. The corresponding improvement means can be used as a further step to explore the way to improve with a higher priority, as a recommendable way of recombinative innovation to better stimulate the thinking of R & D personnel.

Specifically, this paper takes the combination method as the preceding item and the improvement method as the latter item, uses another confidence indicator in the association rules to generate strong association rules, and then uses the promotion degree to further test these filtered rules. By comparing the promotion degree of a specific combination method for different improvement ways, we can generate a relatively higher priority improvement method.

The topic recognition of the patent text by the LDA model can be related to the classification of technical attributes by the TEMPEST model, which will contribute to studying the technical system from the perspective of association because the knowledge elements in the patents are not isolated. Rather, it is a technical entity that is ultimately formed in different dimensions with different coupling ways. Combined with expert opinions and in accordance with the check table of the transformation rule, we can eventually determine the coupling mode of each patent. The reason for this is to examine two important characteristics of knowledge elements: the diversity of knowledge elements and the way they are connected.

2. Priority analysis for improvement direction

Another important feature of recombinative innovation is to examine the relationship between elements, that is, how elements are coupled together. For the analysis of the coupling system, this paper confirms with the help of the SCAMPER model. In this step, the experts’ judgment on the basic rules of the SCAMPER model and related problems is used to determine the coupling mode of each patent element. It is worth noting that, as it is a purely qualitative analysis, to ensure the accuracy of the judgment, opinions from different experts should be taken in to obtain relatively reasonable results.

Answering the rationality of idea generation is the last core question to be discussed in this paper. More recent studies have focused on the methodology itself that generates new and creative ideas, and many of those have stressed the underlying concept that creative ideas are crafted from existing knowledge by identifying novel combinations of previously separated ideas or concepts [9,17,33,53,54]. In other words, a method that can systematically combine valuable knowledge from novel data sources is expected to generate creative ideas. Through the previous exploration of the LDA topic, patent text is mined in two aspects, namely, the multidimensional attributes of elements in patent text and the coupling mode between elements. In this paper, association rules are used to mine the optimal matching pattern between multidimensional knowledge elements and the coupling mode of elements.

Association rule analysis is one of the most active research methods in data mining, which aims to discover the association relations between items not directly represented in the data set [55]. Association rules in a database consist of two different sets of items, called the preceding and the latter [56]. Finally, association rules are used to analyse the strength of association between knowledge elements. Frequent item sets are screened out by setting appropriate confidence and support degrees, and improvement degrees are used as the recommendation basis for R & D personnel to generate ideas.

## 4. Application Case of the Exploitation Technology for CSG

### 4.1. The Phase of Knowledge Element Collection 

#### 4.1.1. Background

The CSG industry is different from other industries. First, there are many influencing factors and a highly dynamic complexity. Second, the portability of exploitation mode is poor, so different storage conditions require different independent innovation technologies. Therefore, the knowledge elements in this technical field are characterized by a multilevel and high coupling, so the reorganization of knowledge elements in this field can often lead to more radical new ideas and provides a reference for the reorganization and innovation of complex technical systems.

#### 4.1.2. Patent Data Collection and Preprocessing

The samples used to execute idea generation come from patents filed by the Chinese intellectual property office and the Derwent database between 2000 and 2018. To define the scope of the target task, the search scope is limited to CSG mining and gas control. At the same time, it is worth noting that patents collected need to be further screened, a step based on thorough expert review. Because the core of this paper is the improvement or innovation of CSG mining methods, those patents related to instrument detection and simulation are not involved in the process of creative generation. In other words, before conducting patent analysis, we remove these patents that do not focus on CSG mining and treatment (the target technical field) for idea generation, such as patents for simulation testing or appearance design, so as to obtain patents related to the target task. Therefore, we can obtain patents more related to new technologies or new ideas. Searching for “CSG mining and treatment” as the keywords, a total of 789 patents were obtained, and the number of patents used for analysis after screening was 378. 

The task of preprocessing focuses on the summary of a target patent, and 378 patent abstracts were preprocessed by tokenization, lemmatization, stop-word removing, and vector-space representation. In this process, Python is used to perform the preprocessing task, whereby the vectorization of the patent abstract can be realized by using the Gensim package in Python. Irrelevant terms that appear in most patents are excluded to further construct the keyword co-occurrence matrix that can reflect technical keywords.

### 4.2. The Phase of Knowledge Element Depth and Diversity Analysis

#### 4.2.1. Technical Topic Extraction by LDA 

In the last step, we process vectorized text data into the LDA algorithm. Using the LDA method, you need to specify the number of topics. As a result of partitioning, the iteration is optimized by adjusting the number of topics. After repeated verification, experimental data show that when the title K is set too low, the text cannot present the title with a clear discrimination ability. Conversely, if you want to identify a very segmented topic, then increase the number of topics; however, if multiple topics are similar, the number of topics will increase. Therefore, the topic clustering of different topics was combined with domain expert opinions to make K = 10. The results of the topic extraction are shown in Table 3.

The parameters of the topic model are set as follows: a = 50/K, k is the number of themes, β=0.01, and the number of iterations is 100. N minus top is 20, that is, the first 20 words of the probability value of each topic are used as the topic words.

As shown in Table 3, the results of the topic clustering are represented by keywords with different probabilities, which are the reflection of technical topics. In the next section, hot spots are identified of technological innovation in CSG mining by knowledge network mapping analysis. Meanwhile, to investigate the diversity of knowledge elements, this paper uses the technical classification system to divide the technical keywords corresponding to the LDA theme into dimensions. Specifically, knowledge elements are divided into different levels according to the TEMPEST model to realize the deconstruction of multidimensional knowledge elements.

#### 4.2.2. Knowledge Map Analysis

**Figure 3:**
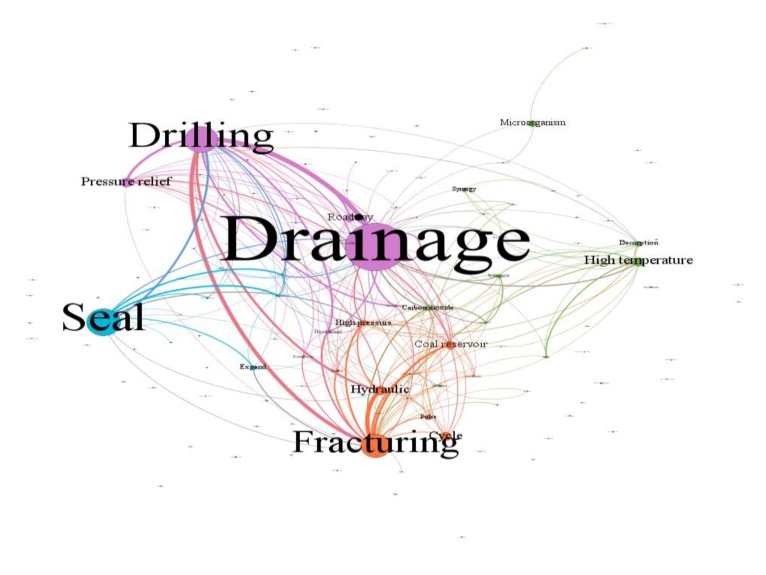
Knowledge network map in the field of CSG.

This paper uses Gephi software to construct the co-occurrence matrix of technical keywords in the target technology field. Through this software, not only can knowledge elements be visualized, but also the attributes of the node itself can be counted. The screening and extraction of keywords is mainly through the IF-IDF text processing of the patent texts previously screened for CSG mining and treatment and finally form a capable co-occurrence matrix of keywords A that reflects the technical characteristics of the field. At the same time, the social network analysis method is applied to the co-word analysis, and the co-word analysis can express the frontier research points as the information metric. Specifically, the keyword can be used as a node. The more central the node is, the more core it is. The relationship between words is represented by the connection between nodes. The thicker the connection is, the closer the relationship or the research structure of the subject. According to the knowledge map, the distribution of the theme of the technical system can be reflected as the standard of subject clustering. We visually analyse the results of the co-word analysis and finally obtain the co-existing network knowledge map of high-frequency keywords in the field of CSG mining and treatment technology, as shown in Figure 3.

The knowledge map of the co-occurrence network in the field of CSG mining technology is shown in Figure 3, which can reflect the distribution characteristics of clustering centres in the different dimensions of knowledge elements in this field. As shown in the Figure 3, we can know by the centrality of a node in the knowledge map (to some extent, it reflects the importance of this node) that the elements of drainage, pressure relief, desorption, seal, drilling, fracturing, hydraulic, high temperature, cycle, coal reservoir, roadway, microorganism, high pressure, carbon dioxide, etc. are the core nodes in this field. These knowledge elements themselves have attribute characteristics in different dimensions. For example, functional elements such as drainage, pressure relief and desorption are the main purposes of innovation in this field, while fracturing, displacement temperature and drilling are important means to achieve the above functions or objectives. Carbon dioxide, nitrogen, proppants, microorganisms, etc is its material base. These interconnected key nodes can reflect that the knowledge elements of a technical system do not exist in isolation but are interconnected and ultimately realize a specific function. It is worth noting that, using social network analysis methods, a number of subject words gathered together can form a research subject area, and mapping network knowledge maps can show the distribution (core and edge) of each research subject under interaction. The knowledge map does not reflect the maturity of the vocabulary (the subject area) well. Therefore, this paper extracts the patent text from the field of CSG mining through the LDA topic extraction model to further analyse the potential information in the patent text.

#### 4.2.3. Technical Topics and Keywords Classification by TEMPEST

**Figure 4:**
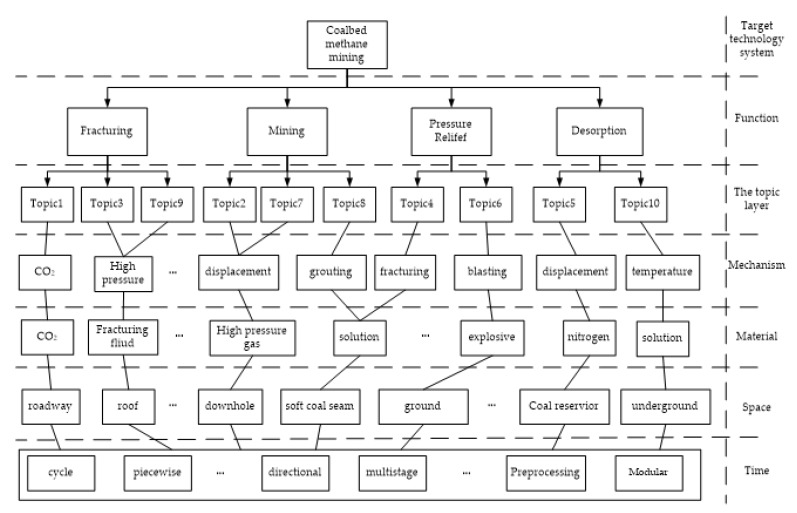
Knowledge network map for the field of CSG mining.

This paper compares the distribution characteristics of the network knowledge map with the results of the LDA topic clustering, and it can be seen from the technical keywords related to the LDA topic that each topic also serves a certain function. In other words, the theme of the LDA extraction reflects the different functional orientations in the field of CSG exploitation technology. It can be further explained that a complete function needs the assistance of knowledge elements of different dimensions to be complete. Based on this, combined with experts in this field and TEMPEST, the innovation dimension of CSG mining technology is determined from topics extracted from LDA. Specifically, the target technology system implements the technical classification through the TEMPEST model. As shown in Table 4, the effect of the classification depends on whether the definition of different attributes in the model is strictly enforced. In this paper, the theme extracted by LDA and a series of corresponding keywords can reflect that the knowledge elements in the field of CSG mining technology are mainly distributed in the dimensions of function (F), mechanism (M), material (M), space (S) and time (T). It should be noted that the different dimensions directly represent the cooperative relationship, rather than the subordination relationship, and the interaction between the knowledge elements of these different dimensions can jointly complete a theme and achieve a specific function. The details are shown in Figure 4.

The multidimensional deconstruction table for the field of CSG mining technology is shown in Table 4, which reflects the field of CSG mining multidimensional coupled knowledge network. The combination of CSG mining technology expertise and the deconstructed LDA topics, the elements of technological innovation are divided into the following different dimensions: space, material, mechanism and functional dimensions. The time level is a reflection of the arrangement of elements in the process and does not contribute to combination, so it is independent. Therefore, the mutual coupling mode of function-material-mechanism-space-time is formed for the field of CSG exploitation. Among them, the spatial dimension involves the layout of CSG mining technology in the spatial structure (including ground, CSG reservoir, surrounding rock, etc.), which reflects the technical implementation direction of knowledge elements in different positions. From the mechanism dimension, the technical characteristics of CSG mining are investigated by changing the working mode of the CSG mining system or the principle of interaction between elements. The technical innovation elements include drilling (u well, cluster well, etc.), fracturing, pulse, etc. Another important dimension of knowledge elements is reflected in materials, including fracturing fluids, proppants, liquid nitrogen, etc. (including chemical materials related to CSG production and their phase states).

After extracting the topic and classifying the dimensions of CSG mining technology, we can clearly understand the distribution characteristics of knowledge elements in this field. More importantly, we can further clarify the correlation of knowledge elements within or among these dimensions. At the same time, through the analysis of the expert opinion, we can have a preliminary understanding about the relationship among the different dimensions. For example, the dimension under the theme of the desorption function is a function of the carbon dioxide and the displacement mechanism via the synergy of implementation. However, the strength of this relationship also needs deeper analysis, so it is necessary, through the association rules, to analyse the relationship between these elements.

### 4.3. The Phase of the Knowledge Element Relationship Analysis

#### 4.3.1. Structure the Patent

This paper needs to explore the relationship between the specific elements of the CSG mining field, so the knowledge elements are projects for correlation analysis. In the same way, on the basis of this, each patent can be understood as a carrier of different projects (knowledge elements). Through the previous analysis of the knowledge elements in the patent text, the knowledge elements of different dimensions in the patent can be identified. In this paper, by comparing the knowledge elements of the different dimensions in Table 4 with each patent, the corresponding combination of knowledge elements in each patent is determined. After determining the combination of knowledge elements corresponding to each patent, these combinations, similar to tags, can reflect the technical characteristics of the corresponding patent to a certain extent, and the patent text can be structured. Next, we need to adjust the coupling of existing knowledge elements in these patents. This step will be analysed through the SCAMPER model, which can adjust the coupling between composition methods in seven different ways to stimulate the generation of new ideas. The combination and improvement directions of the elements corresponding to the patent are shown in Table 5.

As shown in Table 5, a complete technical entity can be expressed as a mutual coupling system formed in different ways among different dimensions. Taking the machine for the patent 3 “CSG linkage hydraulic mining technology system” as an example, analysis of the technical topics and dimension deconstruction, such as for patent 1, reflect the patent knowledge structure as follows. To realize the displacement of CSG in a coal seam, apply the first hydraulic with the synergy of a shock wave in the coal seam and then use nitrogen and carbon dioxide to replace the freed gas. The corresponding operation mode is utilized for multistage segmental operation.

#### 4.3.2. Generate Strong Association Rules

From the previous content, it can be known that the division of knowledge element attributes can realize conceptual layering, that is, the dimensional-factor layer. In the first step, there is a certain correlation between the attributes of knowledge elements. Conceptual layering can be used to generalize the data by replacing lower-level abstract values with higher-level abstract values. Conversely, by replacing higher-level abstract values with lower-level abstract values, conceptual layering can also specialize the data. The attribute divisions of different fine granularity make it possible for association rules to make targeted analysis on the potential connections of knowledge elements in different dimensions. The main content of this paper is to describe the technical system from different dimensions and to extract the correlation between different dimensions and users’ interests and preferences, allowing targeted personalized recommendation.

**Figure 5:**
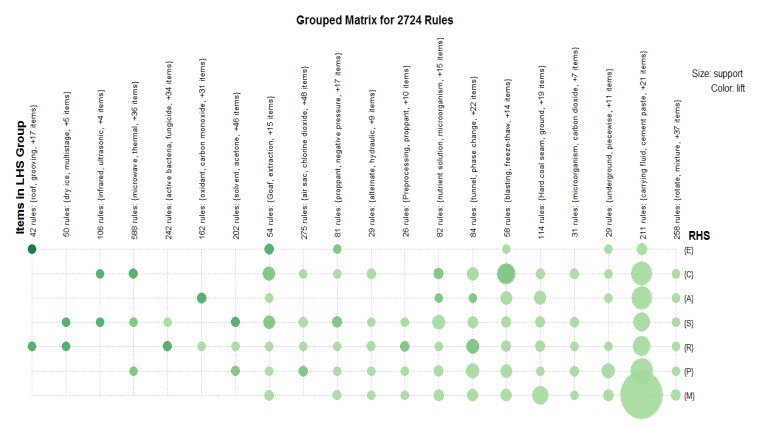
Bubble diagram of the association rule distribution.

Specifically, after topic extraction and exploration, we use two steps to apply the proposed approach. The first step is to extract association rules from the data set, using the Apriori algorithm and using minimum support = 0.10 to extract frequent item sets (see Figure 5). The results are sensitive to the minimum support introduced in the first step of Apriori algorithm. The second step is to generate association rules from the previously extracted set of frequent items. The extracted rules are shown in Table 6. For association rule mining, in this study, we used the arules package in the R tool that is based on the Apriori algorithm, one of the association rule mining algorithms, and provides the infrastructure for representing, operating, and checking transaction data and patterns.

As shown in Table 6, LHS represents the preceding item and LHS represents the latter item. This paper set the different dimensions of the knowledge elements referred to in the preceding item and the improvement directions as the latter item. It is worth noting that the function dimension knowledge of specific elements in the patent and concrete implementation guidance elements are not directly involved in the analysis of association rules. By setting the corresponding support and confidence, the enhance degree can reflect the connection between the factors and the coupling mode intensity. After the analysis to filter out, the total conforms to a total of 2724 sets of association rules, and a bubble diagram reflecting the distribution characteristics of association rules is generated. The execution of this task consists of two components: the Rstudio Server and R packages for association rule mining and visualization.

The distribution of the combination mode and coupling mode of the elements in the field of CSG mining technology is reflected in Figure 5. The size of bubbles in the figure represents the size of the support degree, and the depth of colour represents the degree of lift. The darker the colour is, the higher the lift degree. As shown in Figure 5, in the field of CSG mining technology, there is a strong correlation between the material dimension and mechanism dimension, while in different dimensions, using local optimization, friendliness, and substitution rules to carry out technological innovation has the strongest degree of promotion, which means that experts can fully tap the hidden strong correlation. Some strong association rules are shown in Table 6.

As shown in Table 6, these rules are filtered through three indicators: support, confidence, and lift. These rules provide very clear and useful insights that can be directly applied to the business. From the perspective of knowledge reorganization, different knowledge elements (LHS) reflect the technical characteristics in different dimensions, and the improvement method (RHS) can be used as a way to guide the coupling of different dimensions of knowledge elements, which can help the R & D personnel to analyse and use for knowledge discovery. For example, for rule 3, the new combination forms of knowledge elements are: carbon dioxide, foaming agent and nitrogen in the material dimension can be reorganized in the C (combined) way with the element displacement and high temperature in the mechanism dimension to provide reasonable suggestions for the emergence of creativity.

#### 4.3.3. Priority Analysis of Improvement Direction

We use multicriteria decision support to prioritize the extracted rules. The application of the association rule analysis algorithm will generate a large number of association rules. However, it is difficult to derive useful insights from such a wide range of results. Therefore, it is necessary to compare different improvement directions with the same element combination, to identify the more influential creative generation mode in a specific state, as a recommended option for developers to generate ideas.

Therefore, this paper investigates the association rules among patent knowledge elements in the field of CSG technology and further analyses the 2724 association rules obtained. First, from the 2724 strong association rules, we select the element layer (combination) with high support as the former term (LHS) and the different improvement directions as the preceding item (LHS). On this basis, by comparing the lift of different elements and coupling modes, the most influential coupling mode can be found.

This paper analyses the promotion degree of specific combinations of elements in different improvement directions with the priority order being triggering creativity. As shown in Table 7, for example, for the combination of high pressure, pulse and fracturing, there are different lift degrees in different improvement directions, which can be used as a basis for the priority analysis of element reorganization and innovation. On behalf of the rules, the table blank spaces cannot be produced by setting the threshold value of the corresponding association rules. Therefore, for {High pressure, Pulse, Fracturing → a (Adapt)}, this association rule has a higher priority performance, so it can serve the process of creative generation. It is worth noting that the association rules, as the A recommendation algorithm, also need concrete enforcement of the rules according to the user experience judgment. Therefore, this article, with respect to CSG technology development in the field of creative generation, needs to be combined with domain experts to filter the strong association rules of trade-offs and reprocessing. By using the relationship between the combination of these elements, experts can be directed to restructure the elements of innovation, to generate a detailed description of creativity. Based on the expert experience, the detailed expression of the association rule is obtained to generate new ideas. The specific contents about the idea description are shown in Table 8.

This paper uses the corresponding technical elements of different dimensions to carry out technological innovation. Under the guidance of strong association rules, the identification and recommendation of innovative schemes are carried out to reconstruct the technical elements. Combined with the detailed description of technical opportunities by experts in the field, the technical opportunities for CSG exploitation are identified. It is worth noting that the basis for idea generation in this study comes from the reorganization of the existing knowledge structure in the field of CSG technology. By exploring the correlation strength between the knowledge elements of different attributes, the potential combination patterns of elements and improvement direction with higher priority that have not yet been identified in this field are determined to help experts develop more promising new ideas.

## 5. Conclusions

The exploitation of CSG has different characteristics compared with other industries: diversity, complexity, interactivity, and dynamics. Therefore, how to develop more and efficient exploitation technologies suitable for different coal seams is an important and complex problem for the CSG industry. In view of this, in the process of exploiting CSG, a systematic approach is urgently needed to explore the characteristics of knowledge elements and reconstruct these elements to generate innovative ideas.

In the field of technological innovation, morphological analysis has been regarded as a systematic method of recombinative innovation for generating new ideas. However, in the process of morphological analysis, the recognition and expansion of morphologies often depend on experts’ experience. Meanwhile, in the process of generating creative ideas, a morphology matrix will lead to a large number of redundant combinations without a basis for priority judgment, which will increase the blindness of idea generation. However, previous researches mainly focus on how the quantitative characteristics of knowledge elements affect the innovation results of the target technology and fail to reflect the significant impact among knowledge elements. In view of this, this paper makes some contributions in the field of technology innovation. Firstly, this paper proposes a new method, which can analyze the characteristics of knowledge elements and their relations in the process of technology innovation, and thus provide a more standardized and systematic thinking mode. In other words, from the perspective of recombinative innovation, the process of technological innovation can be structured into three parts such as: the collection of knowledge elements, the diversity and depth analysis of knowledge elements, and the relationship analysis of knowledge elements.

Secondly, this paper takes patents as the carriers of knowledge recombination and identifies the optimization method in the recombinative process through the LDA and association rules, which is expected to help stimulate creative ideas. To be specific, on the one hand, technical topics in patents can be quickly extracted from patent information through topic clustering by LDA model. It is easy to search for key knowledge elements affecting technological innovation according to the technical topics, instead of simply relying on the quantitative characteristics of knowledge elements (e.g., extracting high-frequency words). Combined with the TEMPEST model, this approach provides an automatic generation process for mining deep-level and diverse knowledge elements. On the other hand, in the analysis of the relationship among knowledge elements, this paper combines the SCAMPER model and association rules to analyze the potential relationship among knowledge elements and innovation directions, thereby generating potential strong association rules through the relevant indicators (e.g., support, confidence, lift). This method provides quantitative support to generate creative ideas and helps R & D personnel to overcome a reliance on expert’s experience.

In conclusion, this paper maximizes the effectiveness of the proposed framework by developing automated systems that facilitate the generation of ideas. The use of the proposed model provides practical significance for the use of big data in creative generation and facilitates the rapid decision-making of creative generation. Overall, the recommended process consists of fairly complex steps and many human steps. Therefore, in future research, a simple process and system should be developed to overcome the technical limitations mentioned above and reduce human participation in the process. In addition, the model needs to be validated by conducting more case studies.

## Figures and Tables

**Table 1:** Process comparison of different innovative methods.

		SIT	FA	AD	MA	TRIZ
Dimension layer	Morphological (Elements) analysis			●	●	
	Functional analysis	●	●			●
Generation of ideas	Elements of restructuring					●
	Morphological combination	●			●	

**Table 2:** TEMPEST model.

Analytical Perspective	Description
Function	The functional dimension refers to the ability to solve specific problems and have specific effects in the innovation target system
Material	Stuff comprising the product or technology such as raw materials and ingredients
Time	The sequence dimension refers to the process related to the sequence of time in the innovation objective system
Mechanism	The mechanism dimension refers to the original physical, chemical, biological and other basic principles to realize functions in the innovation target system
Space	Including direction, position, volume, weight, colour and shape

**Table 3:** The results of the LDA analysis (part of the topics).

**Class**	**Keyword**	**Value**	**Class**	**Keyword**	**Value**	**Class**	**Keyword**	**Value**
Topic 1	CSG	0.093	Topic 2	seal	0.399	Topic 3	blasting	0.307
	extract	0.085		pressure	0.087		construction	0.090
	penetration	0.046		inflation	0.082		directional	0.079
	liquid	0.036		extraction	0.043		process	0.073
	roadway	0.034		filling	0.040		fracturing	0.040
	concentration	0.031		crack	0.012		coal seam	0.037
	horizontal	0.029		concentration	0.011		the joint	0.028
	release	0.016		gas	0.011		suction	0.021
	crack	0.011		device	0.010		the release	0.020
	CO2	0.009		location	0.001		hydraulic	0.014
**Class**	**Keyword**	**Value**	**Class**	**Keyword**	**Value**	**Class**	**Keyword**	**Value**
Topic 4	Coal seam	0.088	Topic 5	(CSG)	0.181	Topic 6	drilling	0.005
	extraction	0.068		injection	0.064		drainage	0.005
	downhole	0.054		gas	0.061		blasting	0.005
	mining	0.049		mining	0.056		pressure	0.005
	the ground	0.037		nitrogen	0.031		seal	0.005
	drilling	0.035		CO2	0.028		the mine	0.005
	horizontal	0.020		pressure	0.027		homework	0.005
	fracturing	0.019		system	0.023		coal seam	0.005
	impact	0.017		recovery	0.020		injection	0.005
	pressure relief	0.015		reservoir	0.018		relief	0.005

**Table 4:** The multidimensional deconstruction of the knowledge element table.

Target Technology System	Dimension Layer	Element Layer
	Function	Pressure relief
		Permeability
		Fracturing
		Desorption
		Prevention
		Dust control
		Anti-plugging
		Permeability enhancements
		…
	Mechanism	Thermal
		Nitrogen injection
		Displacement
		Microbial
		High temperature
		Cooling
		Power supply
		Blasting
		High pressure
		Oscillation
		Hydraulic
		…
	Material	Proppant
		CO2
		Nitrogen
		Fluoropolymer
		Microorganism
		Cement paste
		Polyurethane
		Polymer
		Carbon monoxide
		Sealant
		Proppant
		…
	Time	Modular
		Piecewise
		Multistage
		Directional
		Alternate
		Preprocessing
		Synergy
		Multistage
		Multistage
		…
	Space	Underground
		Roadway
		Coal reservoir
		Downhole
		Soft coal seam
		Hard coal seam
		Thick coal seam
		Goaf
		Tunnel

**Table 5:** The combination of knowledge elements corresponding to patent.

TID	Attribute Characteristics and Score of Knowledge Elements					
	Space	Mechanism	Material	Operation mode	Function	SCAMPER
Patent 1	Underground	Shock wave			Fracturing	S (substitute)
Patent 2	Hard coal seam	Blast			Pressure relief	S
Patent 3		Waterpower shock wave	Nitrogen, Carbon dioxide	Multistage	Displacement	C (combine)

**Table 6:** Association rule table.

	LHS (the Preceding Item)	RHS (the Latter Item)	Parameter		
			Support	Confidence	Lift
Rule 1	Coal reservoir (Space), Fracturing (Mechanism), High pressure (Mechanism), Nitrogen (Material)	E (Eliminate)	0.004	1	17.714
Rule 2	Fracturing (Mechanism), Fracturing fluid (Material), Multistage (Time)	R (Rearrange)	0.008	1	8.857
Rule 3	Carbon dioxide (Material), Displacement (Mechanism), High temperature (Mechanism), Foaming agent (Material), Nitrogen (Material)	C (Combined)	0.004	1	8.266
Rule 4	Desorption (Mechanism), Directional (Time), Expand (Personality)	P (Put to other uses)	0.004	1	5.904

**Table 7:** The lift degree under different association rules.

LHS		RHS						
		A	S	C	P	M	E	R
High pressure, Pulse	Fracturing	8.86		8.23		3.1		
Nitrogen, Carbon dioxide	Displacement/Drainage			8.26	5.9			8.86
High temperature	Desorption/Displacement/Drainage	8.86	7.76	8.26	5.90	3.1		8.86
Waterpower, Drilling	Fracturing			4.13		1.16		1.11

**Table 8:** Idea description.

Space	Mechanism	Material	Rule	Idea Description
Coal reservoir	High pressure, Pulse	Liquid nitrogen	A (Adapt)	Since liquid nitrogen (M) has a good sand-carrying performance and clear stimulation effect, it can be used to remove plugging and support existing fractures and optimize (A) fracture conductivity, to increase gas production through repeated fracturing
	Microbial desorption + fracturing	Fracturing fluid	M (Modify, Mingy)	The fracturing fluid used in coal seam fracturing is the biological fracturing fluid mixed with hydrogen-producing bacteria and methanogenic bacteria. Hydrogen-producing bacteria and methanogenic bacteria can improve the desorption ability of coal seam without harming coal seam
	Drilling		Replace	The use of cutting instead of drilling can greatly increase the area of a single action, greatly improve the construction efficiency and reduce the amount of engineering and construction cost
